# Neuropathic Pain in Animal Models of Nervous System Autoimmune Diseases

**DOI:** 10.1155/2013/298326

**Published:** 2013-05-08

**Authors:** David H. Tian, Chamini J. Perera, Gila Moalem-Taylor

**Affiliations:** School of Medical Sciences, University of New South Wales, Sydney, NSW 2052, Australia

## Abstract

Neuropathic pain is a frequent chronic presentation in autoimmune diseases of the nervous system, such as multiple sclerosis (MS) and Guillain-Barre syndrome (GBS), causing significant individual disablement and suffering. Animal models of experimental autoimmune encephalomyelitis (EAE) and experimental autoimmune neuritis (EAN) mimic many aspects of MS and GBS, respectively, and are well suited to study the pathophysiology of these autoimmune diseases. However, while much attention has been devoted to curative options, research into neuropathic pain mechanisms and relief has been somewhat lacking. Recent studies have demonstrated a variety of sensory abnormalities in different EAE and EAN models, which enable investigations of behavioural changes, underlying mechanisms, and potential pharmacotherapies for neuropathic pain associated with these diseases. This review examines the symptoms, mechanisms, and clinical therapeutic options in these conditions and highlights the value of EAE and EAN animal models for the study of neuropathic pain in MS and GBS.

## 1. Introduction

Neuropathic pain is caused by a lesion or disease of the somatosensory nervous system either at the peripheral or central level and is a frequent presentation in a myriad of medical conditions [1]. It is characterised by abnormal sensations or hypersensitivity in the affected area, which is often combined with, or is adjacent to, areas of sensory deficit [2]. Symptoms include tactile or thermal hypoaesthesia (reduced sensation to nonpainful stimuli), hypoalgesia (reduced sensation to painful stimuli), loss of sensation, paraesthesia (abnormal sensations such as skin crawling or tingling), paroxysmal pain (e.g., shooting, electric shock-like sensations), spontaneous ongoing pain (not induced by stimulus e.g., burning sensation), and evoked pain (i.e., stimulus-induced pain), the last of which includes hyperalgesia (increased sensitivity to painful stimuli) and allodynia (perception of innocuous/non-painful stimuli as painful) [2]. In particular, neuropathic pain is common in autoimmune demyelinating diseases of the nervous system, such as multiple sclerosis (MS) and Guillain-Barre syndrome (GBS), and adversely affects millions of sufferers worldwide [3, 4].

Thus far, several animal models have been established to mimic features of MS and GBS, so as to better enable researchers to understand the underlying pathophysiology and immune mechanisms and to investigate better therapeutic options. For example, experimental autoimmune encephalomyelitis (EAE) serves as the classic animal model of multiple sclerosis, whereas experimental autoimmune neuritis (EAN) mimics acute inflammatory demyelinating polyneuropathy, the most common subtype of GBS [5–7]. These two models are the most widely used and accepted analogues of MS and GBS and provide many immunological parallels. In this review, we discuss the symptoms, mechanisms, and potential therapeutic strategies in neuropathic pain associated with EAE and EAN.

## 2. Multiple Sclerosis and Experimental Autoimmune Encephalomyelitis

Multiple sclerosis is a chronic, T-cell mediated autoimmune inflammatory disease of the central nervous system (CNS) that predominantly affects the myelin sheath. It is the most common cause of acquired disability in young adults in the western world [8–10]. Among the many sensory disturbances present in MS, pain—nociceptive, neuropathic, or mixed—is a highly prevalent symptom, reported by 25 to 90% of patients [3, 11–17]. It negatively impacts on general health, energy and vitality, mental health, and social functioning [17, 18], as well as impinges on daily life [12, 14, 19]. Despite its prevalence, the specific underlying mechanisms of MS pain are still not well understood [20], although elucidation has been sought through recent studies in animal models [21–23].

Experimental autoimmune encephalomyelitis has frequently served as an animal model of MS. EAE is commonly induced in genetically susceptible animal strains by immunisation with a self-antigenic epitope of myelin, which causes characteristic breakdown of the blood-brain barrier and multifocal infiltration of activated immune cells that attack the myelin sheath [8]. The ensuing immunologic response leads to chronic neuroinflammation, demyelination, and neuronal damage in the CNS. The species-specific disease course exhibits close clinical and histopathological similarities to various forms of MS [24–26], thereby presenting EAE as a suitable model to study multiple sclerosis [27, 28].

## 3. Symptoms of Neuropathic Pain in MS and EAE

MS patients often experience a wide range of neuropathic pain symptoms. This includes ongoing extremity pain (characterised by constant pain in the legs and feet), trigeminal neuralgia (characterised by paroxysmal attacks of electric-shock-like sensations in specific facial or intraoral areas), Lhermitte's phenomenon (characterised by a transient electrical sensation that runs down the back and is related to neck movement), and thermal and mechanical sensory abnormalities [18, 29]. As behavioural models of ongoing extremity pain and paroxysmal pain in animals are currently unavailable, most animal studies have focused on thermal and mechanical abnormalities (Table 1). EAE has thus far served as the basis for preclinical research into the mechanisms of these abnormalities. Genetic, clinical, and histopathological heterogeneities of EAE models produce different sensory and pathological changes, allowing for robust representation of the various forms of pain in MS [30].

### 3.1. Heat Disturbances

In multiple sclerosis, neuroinflammatory lesions in the CNS produce significant somatosensory deficits, particularly for temperature discrimination, such as paradoxical heat sensations and altered heat/cold thresholds [38–40]. Up to 58% of MS patients have reported suffering from heat sensitivities, which is a significant cause of fatigue, concentration problems, and pain [41]. Such abnormalities have been paralleled in several EAE studies.

In an early study, Duckers and associates noted 23%–58% prolongation of reaction time to noxious heat at 10 and 18 weeks following EAE induction in Lewis rats, suggestive of chronic hypoalgesia [31]. More recently, Aicher and colleagues observed a dynamic thermal response in a chronic relapsing-remitting form of EAE induced by a myelin proteolipid protein (PLP). In both male and female SJL mice, initial thermal hypoalgesia occurred concurrent with onset of clinical symptoms, later manifesting as chronic thermal hyperalgesia of the tail. Hyperalgesia was more sustained in female mice [21], reflecting a sex-linked disease profile [42]. While the magnitude and duration of tail hyperalgesia were seen to be related to the severity of motor symptoms, thus potentially cofounding the results, it was noted that the onset of hypoalgesic nociceptive responses preceded motor dysfunctions by several days [21].

Similar studies in chronic EAE induced in C57BL/6 mice with myelin oligodendrocyte glycoprotein (MOG) also reported heat hypoalgesia in the hindpaws, developing subsequent to symptomatic onset of disease, although this was also theorised by the investigators to be affected by concurrent gross locomotor disabilities [22]. In contrast, thermal hyperalgesia in the hindpaws developed during the chronic disease phase in SJL and C57BL/6 mice immunised with PLP or MOG, respectively [30]. In a study of both acute and chronic EAE induced by myelin basic protein (MBP) in rats, comparable tail heat allodynia was reported, with the onset of thermal abnormalities appearing prior to the development of clinical signs [23].

These findings show differential thermal responses and concur with case reports of heat hypoalgesia [43], as well as thermal hyperalgesia [18, 44, 45] in MS patients.

### 3.2. Cold Disturbances

Cold allodynia, a reported sensory disturbance in MS patients [18], has been observed in several EAE models. In particular, cold allodynia in response to application of acetone to the hindpaws has been demonstrated in mice with a MOG-induced chronic-relapsing EAE prior to and during onset of motor disturbances [22]. Similarly, cold sensitivity at the level of the hindpaws was noted in EAE rats that were tested on a cold plate, starting before and lasting during and after clinical signs [23]. In the latter investigation, cold hyperalgesia at the level of the tail was also observed, although this was only present prior to clinical onset of EAE. Thibault and colleagues also detected no significant differences in cold allodynia and hyperalgesia between both acute and chronic EAE models, suggesting that abnormalities to cold sensitivities are independent of EAE phenotype [23]. The early onset of cold allodynia parallels the observation that neuropathic pain in MS patients often precedes or is present at clinical onset [11].

### 3.3. Mechanical Disturbances

In addition to thermal abnormalities, MS patients often experience tactile allodynia [11, 18, 46]. For example, both tactile hypoesthesia (reduced sensation to touch) and allodynia have been reported in relapsing remitting forms of MS [45, 47, 48]. A recent study reported high prevalence of hypoesthesia and hyperesthesia (61% and 34%) in patients with MS and central neuropathic pain, although this was similar to a control group of MS patients with painless sensory symptoms [40]. 

In chronic relapsing EAE models (using MOG_35–55_ in mice), robust mechanical allodynia became apparent prior to clinical signs [22], although the response times to tactile stimuli increased during disease peak (hypoalgesia), and reduced following partial amelioration of motor dysfunction. Again, this suggests confounding influence of mechanical paralysis. Similar studies using the same encephalitogenic antigen (MOG_35–55_) elicited comparable results of hypernociception [33, 36], although without hypoalgesia at disease peak. A recent study has demonstrated that the development of mechanical sensitivity is dependent upon the EAE model used; whereas SJL mice immunised with MOG developed marked mechanical allodynia during the chronic phase of the disease, C57BL/6 mice immunised with PLP developed only minor mechanical allodynia during disease onset and peak phases [30].

The robust nociceptive changes were similarly observed in a study using a rat model of MOG-induced EAE, showing periods of both decreased sensitivity to touch prior to the onset of hindlimb paralysis and increased sensitivity to touch (mechanical allodynia) during symptomatic remission [35]. Interestingly, a study using 2 doses of MOG in rats established that a 12.5% reduction in the dosage of the encephalitogenic peptide was sufficient to significantly ameliorate motor deficit profiles but did not significantly alter the robust pain states, thereby highlighting the partial independence of evoked pain presentations to motor dysfunctions [37]. This is a concept previously established through a novel study, whereby the investigator observed the absence of vocalised pain response despite noxious mechanical stimulation of the paralysed tail [32]. As vocalisation reflex can occur unhindered by tail paralysis, it can be surmised that motor paralysis (in this case, of the tail) is not the sole cause of diminished pain behaviours.

## 4. Potential Mechanisms of Neuropathic Pain in MS and EAE

As the importance of pain as a functional disability of multiple sclerosis has only recently been recognised, a clear understanding of its pathogenesis is still absent. Several theories exist to explain its mechanism, including lesions of CNS areas that process pain information, generation of enhanced response to painful stimuli due to loss of descending inhibitory nociceptive pathways, damage to somatosensory nerves, and inflammation of the spinal cord [10, 21, 29, 49].

Some of the proposed mechanisms of neuropathic pain in MS patients include thalamic or cortical deafferentation due to multiple lesions along the spinothalamocortical pathways generating ongoing extremity pain, high-frequency ectopic discharges due to demyelination of the trigeminal afferents producing symptoms of trigeminal neuralgia, and high-frequency ectopic discharges due to demyelination of the dorsal column primary afferents causing Lhermitte's phenomenon [10]. While these mechanisms have not yet been validated through animal studies, preclinical studies suggest that inflammation and gliosis are key mediators in changes in sensory functions (such as cold and tactile allodynia) seen in EAE.

It is well accepted that inflammatory cells and immune-like glial cells and their mediators facilitate central sensitisation and contribute to neuropathic pain symptoms [50]. Indeed, a recent study has shown that animals with EAE did not have altered expression of sensory neuropeptides but had a significant influx of CD3+ T cells and increased astrocyte and microglia/macrophage reactivity in the superficial dorsal horn of the spinal cord, an area associated with pain processing [22]. Furthermore, a significant increase in the level of tumour necrosis factor *α* (TNF) expression in the dorsal root ganglia (DRG) of EAE animals was found at disease peak [51]. A later study confirmed a correlation between the increase in TNF gene and protein expression in the DRG and spinal cord with the onset of neuropathic pain in rats with EAE [52]. Similar increases in the gene expression of cytokines interleukin (IL)-1*β* and IL-6 in the spinal cords of EAE mice coincided with increased nociceptive sensitivity and deficits in object recognition [53]. Gene therapy with anti-inflammatory IL-10 in animals with EAE improved motor and sensory function, prevented allodynia, and reduced glial activation in the lumbar spinal cord [35].

Further mechanisms have implicated the accumulation of infiltrating macrophages expressing purinergic P2X_4_ receptors (P2X_4_R) in CNS lesions of EAE animals [54]. As activation of these receptors by adenosine triphosphate is implicated in the microglial response to peripheral nerve injury and neuropathic pain symptoms, an association between P2X_4_R and neuropathic pain in EAE is suggested [55]. Additionally, increased phosphorylation of transcription factor cyclic AMP response element-binding protein (CREB) has also been observed at disease peak in EAE lesions, particularly in the dorsal horn sensory neurons [56], which are associated with the generation and maintenance of neuropathic pain. Similar involvement of chemokines in leukocyte recruitment, immune regulation, and T-cell polarisation is believed to significantly impact on pain regulation. For example, CCL2, a chemokine with elevated levels in MS patients, amplifies inflammatory responses in EAE [57, 58], while intrathecal administration of CCL2 chemokine is sufficient to induce mechanical allodynia in naïve animals but not in CCL2-receptor knockout mice [59–61].

Dysregulation of the glutamatergic system, caused by reduced glutamate transporter expression in spinal cords, has been implicated in abnormal pain sensitivity in mice with MOG-induced EAE. For example, EAE mice showed a lack of behavioural response to formalin stimulation, a behavioural model of injury-induced central sensitization. This hyporesponsiveness was attributed to a decreased expression of the glutamate transporters EAAT-1 and EAAT-2 in the spinal cord [34]. Furthermore, pharmacological treatment to upregulate the levels of EAAT-2 in mice with EAE resulted in prevention of tactile hypersensitivity and normalisation of performance in cognitive assays [53].

## 5. Clinical Applications of EAE Neuropathic Pain Models

EAE has proven to be a successful therapeutic preclinical model for MS. Indeed, a number of approved drugs and current phase II and III trials for MS were first examined in EAE models [62].

Several pharmacotherapies used to treat pain in multiple sclerosis have shown similar efficacies in EAE. For example, Gabapentin, a *γ*-aminobutyric acid (GABA) analogue used by up to 19% of MS sufferers [63, 64], is highly effective in ameliorating pain symptoms in MS, such as trigeminal neuralgia and tonic spasms [65–68]. Moreover, using Gabapentin, Thibault and colleagues demonstrated a significant reduction of mechanical hyperalgesia in EAE murine models, highlighting the effectiveness of GABA analogues and their therapeutic potentials on neuropathic pain in EAE models [23].

There also exist several promising avenues of pharmaceutical research. Lisi and associates have established that prophylactic Rapamycin administration, a macrocyclic antibiotic with immunosuppressive activity, is able to reduce disease severity and ameliorate pain behaviour in EAE animals [36], confirming similar rodent studies [69–71]. It is theorised that by regulating effector T cell and regulatory T-cell function [72], Rapamycin is able to modulate cytokine release, particularly interferon (IFN)-*γ*  [73], a potent cytokine implicated in neuropathic pain [50].

Another promising candidate for MS pain amelioration targets glutamate transporters. MS patients are known to have an elevated concentration and/or altered transport of glutamate in the CNS [74–78], partly due to glutamate released by invading T cells and macrophages [79, 80]. This increases extracellular accumulation of glutamate through the downregulation of glutamate transporters and impairment of glial glutamate uptake [81]. The excess glutamate concentrations allow for prolongation of calcium-permeable ionotropic glutamate receptor activation on neural and glial cells, leading to excitotoxic CNS tissue damage [82, 83]. Studies in EAE rodent models have demonstrated 50% reduction in glial glutamate transporter (GLT-1) spinal expression compared to normal animals [37, 84]. In chronic EAE models, administration of ceftriaxone, a third-generation cephalosporin antibiotic which upregulates CNS glutamate transporters, has not only shown to limit and attenuate clinical symptoms [37, 85] but also shown to significantly reverse tactile allodynia [37] and normalise facets of cognitive functioning [53]. Normalisation of pain behaviour has been confirmed using other compounds known to promote glutamate transporter activity in EAE models, such as MS-153 [34].

As MS is a predominantly proinflammatory disease, anti-inflammatory agents predictably demonstrate significant therapeutic potential. Currently, several drugs exist that effectively target the inflammatory process in MS patients [86–89]. In EAE, lumbar intrathecal injections of a plasmid DNA with mutated IL-10 gene, designed to stimulate an anti-inflammatory response, reduced disease course and prevented mechanical allodynia [35]. Furthermore, FTY720, a sphingosine 1-phosphate receptor modulator, has been shown to suppress EAE development in several rodent models [90–93] by reducing the infiltration of CD4+ T cells, macrophages, and proinflammatory cytokines [93–96], as well as by modulating signalling pathways on glial cells [97, 98]. In addition to confining lymphocytes to lymphoid tissue [94] and preventing and reversing pathological disturbances to pre- and postsynaptic glutamate transmission [99], FTY720 is thought to induce endogenous repair mechanisms in the CNS, as it preferentially localises to myelin sheath [100]. Clinically, FTY720 has reduced MS relapse rates and lesion frequency [101–103]. While these studies focus on disease amelioration, Balatoni and associates have demonstrated in a chronic EAE model that prophylactic application of FTY720 prevented evoked potential disturbances of the somatosensory system [104], raising the possibility of using FTY720 to modulate neuropathic pain. In support of this, a recent study has shown that administration of FTY720 reduces mechanical and thermal allodynia in animals with neuropathic pain caused by peripheral nerve injury [105].

## 6. Guillain-Barre Syndrome and Experimental Autoimmune Neuritis

Guillain-Barre syndrome is the most common acute inflammatory demyelinating neuropathy in the peripheral nervous system (PNS), and as such can almost be considered a counterpart to multiple sclerosis. It affects 1-2 individuals per 100,000, with a greater disposition towards men [106]. GBS is a common cause of neuromuscular paralysis, characterised by areflexia or acute hyperreflexia, and can be effectively treated with immunotherapies such as intravenous immunoglobulin. However, despite immunotherapy, GBS has a 5% mortality rate, with up to 20% of patients remaining severely disabled [107]. Other symptoms of GBS include sensory impairments, such as moderate to severe nociceptive and neuropathic pain [4, 108, 109]. In fact, pain is a highly prevalent symptom, with 55–85% of sufferers complaining of paraesthesia/dysaesthesia, backache and sciatica, neck pain, muscle pain, joint pain, and visceral pain [4, 109].

Experimental autoimmune neuritis is a T-cell-mediated acute demyelinating inflammatory disease of the PNS widely used as an animal model of the acute inflammatory demyelinating polyneuropathy, the most common form of GBS [110]. First successfully induced in rabbits by Waksman and Adams in 1955, EAN is characterised by degeneration of myelin sheaths, proliferation of histiocytes, breakdown of blood-nerve barrier, and localised PNS inflammation with infiltration of lymphocytic and mononuclear cells [6].

EAN can be induced by immunisation with neuritogenic peripheral nerve myelin components, purified myelin proteins (such as P0, P2, or PMP-22), or synthetic peptides of myelin proteins [111, 112], or by passive transfer of T cells sensitised to these proteins. Susceptible animals (such as rats, mice, rabbits, and guinea pigs) induced with EAN develop monophasic disease characterised by weight loss, ascending progressive paralysis, and spontaneous recovery.

## 7. Symptoms of Neuropathic Pain in GBS and EAN

Neuropathic pain, primarily affecting the distal extremities, represents a common and severe symptom in patients with GBS and is more common and persistent than nonneuropathic pain [113]. Dysaesthetic extremity pain, described as burning, tingling, or shock-like sensations, has been reported in up to 49% of GBS patients [109]. GBS patients also experience altered thermal sensations, with significantly higher warm threshold temperatures and lower cold threshold temperatures as compared to age- and gender-matched controls [114]. In support of this, a recent study has shown that GBS patients have a significantly more severe impairment of cold detection thresholds, heat pain thresholds, and responses to suprathreshold heat stimuli in the foot, as compared to patients with nonneuropathic pain or without pain [113]. In addition, GBS patients suffer from brush-induced allodynia [113].

The thermal and tactile sensory abnormalities evident in GBS are reflected in EAN models (Table 2). Behavioural tests of pain hypersensitivity in EAN, including thermal hyperalgesia and mechanical allodynia, have frequently served as tools to study GBS sensory dysfunctions. For example, Moalem-Taylor and colleagues were able to observe significant mechanical allodynia and thermal hyperalgesia in both hindpaws and forepaws of rats with EAN [115]. A subsequent study confirmed the development of neuropathic pain in EAN animals and further demonstrated that mechanical allodynia preceded the onset of neurological signs and persisted after cessation of locomotor deficit [116].

## 8. Potential Mechanisms of Neuropathic Pain in GBS and EAN

Despite its prevalence, the mechanisms of neuropathic pain in GBS patients remain unknown. It has been suggested that in the acute phase of GBS, neuropathic pain results from nerve inflammation, whereas in the chronic phase of the disease, neuropathic pain results from degeneration of sensory nerve fibres [121]. Recently, it has been shown that a considerable reduction in intraepidermal nerve fibre density at the distal leg is evident early in the disease and correlates with pain intensity in the acute phase of GBS [122]. Furthermore, impairment of small myelinated and unmyelinated nociceptive fibres is significantly greater in GBS patients with neuropathic pain than in those without neuropathic pain. The severity of such impairment during the acute phase of GBS is predictive of chronic neuropathic pain [113].

To date, very few research laboratories have studied the mechanisms underlying neuropathic pain in EAN animals. However, existing studies have implicated several inflammatory mediators and cells in the initiation and maintenance of neuropathic pain in EAN through secretion of inflammatory mediators that sensitise nociceptors to amplify pain hypersensitivity. For example, greater numbers of T cells, antigen-presenting cells, and macrophages were observed in peripheral nerves of EAN animals [115]. These infiltrating leukocytes in the PNS may play a role in EAN-induced pain by releasing proinflammatory cytokines such as IL-18 (an IFN-*γ* inducing factor, produced by macrophages) with significantly greater IL-18 expression observed in nerve roots of EAN rats and significantly higher serum levels of IL-18 detected in GBS patients as compared to control subjects [123]. Cells immunoreactive for inducible nitric oxide synthase and TNF have been also observed in the DRGs of animals with EAN [124].

Additionally, there exists accumulating evidence that microglia become activated following PNS damage and contribute to sensitisation of central nociceptors through the production of proinflammatory cytokines, chemokines, and extracellular proteases [50]. Indeed, an increase in the number of microglial cells has been demonstrated in rats with EAN [116, 120]. In particular, the association of the time course of mechanical allodynia and spinal upregulation of P2X_4_R on spinal microglia in lumbar dorsal horns in EAN rats has been successfully observed by Zhang and colleagues [116]. This suggests that activation of P2X_4_R drives the release of brain-derived neurotrophic factor from spinal microglia, a cellular substrate that causes disinhibition of pain-transmitting spinal lamina I neurons and mediates aberrant nociceptive processing in the spinal cord [125]. The involvement of transmembrane chemokines such as CX3CL1 (fractalkine) has also been implicated, as it plays a key role in mediating neuron-microglia interactions in nociceptive transmission. Elevated levels of CX3CL1 have been recorded in GBS patients [126], while in EAN rats, extensive upregulation of immunoreactivity for CX3CL1 and its receptor CX3CR1 in the dorsal horn has been shown to correlate with the establishment of mechanical allodynia [120].

Taken together, development and maintenance of neuropathic pain in EAN models may result from (a) demyelination and degeneration of sensory nerve fibres, (b) autoimmune inflammation in the PNS, and (c) spinal glial activation in the CNS, therefore providing a useful model for finding novel therapeutic approaches for GBS-related pain.

## 9. Clinical Applications of EAN Neuropathic Pain Models

Although the studies on pain in EAN are inadequate to date, there are a few significant approaches that may be therapeutic in relieving pain in GBS patients.

Firstly, immunotherapeutic approaches that enhance the numbers of immunosuppressive FoxP3^+^ regulatory T (Treg) cells and decrease neuroinflammation have demonstrated potential. Recently, it has been shown that treatment with CD28 superagonist, a Treg cell expander, resulted in a significant amelioration of EAN severity and mechanical allodynia, with associated reduction of neuroinflammatory responses [119]. Treatment with Compound A, a plant-derived ligand of glucocorticoid receptors that enhances Treg cells in blood of EAN animals, is also able to attenuate mechanical allodynia [118]. The same study further observed that Compound A reduced microglial activation and IL-1*β* and TNF upregulation in the spinal cord, increased the numbers of anti-inflammatory M2 macrophages in sciatic nerves, and modulated lymphoid cytokines to an anti-inflammatory profile [118].

Furthermore, statins, which are used to treat hypercholesterolaemia in humans, are reported to potentiate anti-inflammatory and immunomodulatory effects, including deviation of helper T cell Type 1 (Th1) mediated proinflammatory response to Th2 mediated anti-inflammatory response, inhibition of Th1 and Th17 mediated autoimmune response, inhibition of maturation and activation of antigen presenting cells, and increasing the numbers of CD4^+^CD25^+^FoxP3^+^ Treg cells [127]. Recent studies have found that administration of atorvastatin reduces EAN severity through a similar mechanism [127], while treatment with rosuvastatin and simvastatin prevents the development of thermal hyperalgesia and mechanical allodynia and significantly reduces spinal glial activation following peripheral nerve injury [128]. This highlights the potential for statins to manage neuropathic pain in GBS.

In addition, the existing evidence for the role of microglia in neuropathic pain suggests that controlling spinal glial activation may result in pain amelioration in GBS. In particular, inhibition of microglial activation and alleviation of mechanical allodynia has been successfully observed by peritoneal administration of minocycline, which attenuates TNF and decreases proinflammatory cytokine response [117, 129]. Elevated levels of CX3CL1 in GBS suggest that inhibiting CX3CL1/CX3CR1 interactions will negatively affect microglial activation [120] and might prevent the development of neuropathic pain.

Other possible therapeutic approaches include inhibiting matrix metalloproteinases (MMPs). MMPs comprise a large family of proteases that have been implicated in the generation of neuroinflammation and the development of neuropathic pain through the cleavage of extracellular matrix proteins, cytokines, and chemokines [130]. MMP-9 and MMP-7 were found to be selectively upregulated during EAN and expressed in nerves of GBS patients [131]. BB-1101, a broad spectrum MMP inhibitor, has already demonstrated potential in preventing the development of EAN [130], and reduced expression of MMP-9 by treatment with minocycline was associated with improved EAN outcome and reduced mechanical allodynia [117].

## 10. Summary

Although much has been uncovered in the past few decades about the nervous system autoimmune disorders of MS and GBS, the clear pathogenesis of these diseases has not been fully elucidated. However, utilisation of animal models, in particular EAE and EAN, has significantly advanced our understanding and provided a platform for development and investigation of new therapies. Recently it has become clear that neuropathic pain is a common debilitating symptom in MS and GBS and that some of the changes in pain sensitivity observed in these patients can be mimicked in EAE and EAN animals. Tables 1 and 2 summarise neuropathic pain symptoms observed in EAE and EAN, respectively, and the therapeutic agents tested in these animal models. Many complex mechanisms are involved in mediating the various sensory changes, and we are only now beginning to understand the mechanisms underlying neuropathic pain in MS and GBS. A recent study in humans has demonstrated an autoimmune basis for some types of chronic idiopathic pain highlighting the role of autoimmune antibodies and cells in pain mediation [132]. A concerted effort is required to elicit more information regarding the mechanisms underlying neuropathic pain in MS and GBS to better enable the development of more effective treatments.

## Figures and Tables

**Table 1:** Summary of pain symptoms observed in EAE models and potential therapeutic options for treating MS pain.

EAE	Antigen	Animal model	Test site	Test	Symptoms during disease	Drug	Effects of drug	References
			Tail	Water bath immersion	Heat allodynia (before clinical signs and after resolution)^1^			
					Cold hyperalgesia^2^ (before clinical signs)			
Acute	500 *μ*g MBP	♀ 5 wk Lewis rats	Hindpaw	Cold plate analgesia meter	Cold allodynia^3^			[23]
					Cold hyperalgesia^3^ (during clinical signs)			
				Pinch test	Mechanical hyperalgesia (after emergence of clinical signs)			
				Electronic von Frey	No significant difference			

			Tail	Water bath immersion	Heat allodynia (before clinical signs and after resolution)^1^	Gabapentin	Reduce mechanical hyperalgesia	[23]
					Cold hyperalgesia (before clinical signs)^2^			
			Hindpaw	Cold plate analgesia meter	Cold allodynia^3^	Tramadol	Reduce thermal and mechanical hyperalgesia; reduce cold allodynia; known analgesics	
CREAE	500 *μ*g MBP+ Cyclosporine A	5 wk ♀ Lewis rats						
					Cold hyperalgesia^3^ (during recovery)			
				Pinch test	Mechanical hyperalgesia (at disease peak and recovery)			
				Electronic von Frey	Mechanical allodynia (after clinical signs)	Duloxetine	Reduce thermal hyperalgesia; reduce cold allodynia	

CEAE	100 *μ*g GPSCH	♀ Lewis rats	Paws	Hot plate	23% prolonged reaction to noxious heat (hypoalgesia @ wk 10)^4^ 58% prolonged reaction to noxious heat (hypoalgesia @ wk 23)^4^	ACTH4-9^5^	Reduce neurological signs and facilitate recovery of sensory function	[31]

				Hargreaves test	Heat hypoalgesia at disease peak and recovery			
CREAE	50 *μ*g MOG_35–55_	♀ Lewis rats	Hindpaw	Acetone application	Cold allodynia before clinical signs and at disease onset			[22]
				Manual von Frey	Tactile allodynia at disease onset			

CREAE	50 *μ*g GPMBP	Lewis rats	Tail	Vocalisation to noxious mechanical stimuli	Ascending pattern of absent vocalisation			[32]

CREAE	150 *μ*g PLP_139–151_	M/F SJL mice	Tail	Hargreaves test	Heat hypoalgesia at disease peak followed by heat hyperalgesia at disease recovery			[21]

CREAE	40 × 10^6^ T cell blasts^6^	♀ SJL mice	Tail	Hargreaves test	Heat hypoalgesia at disease peak followed by heat hyperalgesia at disease recovery			[21]

CREAE	100 *μ*g MOG_35–55_	♀ C56BL/6J mice	Hindpaw	Electronic von Frey	Mechanical allodynia before clinical signs			[33]

CREAE	50 *μ*g MOG_35–55_	♀ C56BL/6J mice	Hindpaw	Formalin injection	Hyponociception before clinical signs			[34]

RREAE	35 *μ*g MOG_1–125_	♂ Dark Agouti rats	Hindpaw	Manual von Frey	Mechanical allodynia (before clinical signs and during recovery)	IL-10 gene therapy^7^ (plasmid DNA)	Simulates anti-inflammatory response Significant decrease of disease severity Prevention of mechanical allodynia	[35]

CEAE	300 *μ*g MOG_35–55_	♀ C56BL/6J mice	Paw	Manual von Frey	Mechanical allodynia (during disease course)	Rapamycin^8^	Blocks cytokine-driven T-cell proliferation Reduced EAE development Inhibition of mechanical allodynia after development of clinical signs	[36]

RREAE	8.75 *μ*g MOG_35–55_	♂ Dark Agouti rats	Paw	Manual von Frey	Mechanical allodynia (at disease onset)	Ceftriaxone^9^	Upregulates glutamate transporter to reduce excess intracellular glutamate and excitotoxicity Significant decrease of disease severity Reverse tactile allodynia	[37]

CEAE: chronic experimental autoimmune encephalomyelitis; CREAE: chronic relapsing experimental autoimmune encephalomyelitis; RREAE: relapsing remitting experimental autoimmune encephalomyelitis; MBP: myelin basic peptide; MOG: myelin oligodendrocyte glycoprotein; PLP: proteolipid peptide; GPSCH: guinea pig spinal cell homogenate.

^1^42°C; ^2^2°C; ^3^cooled from 22°C to 0°C, demonstrating hyperalgesic or allodynic nociceptive behaviour; ^4^54°C; ^5^repeated s.c. injections of ACTH4-9 peptide; ^6^passive immunisation with transfer of PLP_139–151_ specific splenocytes from EAE-induced animals; ^7^intrathecal plasmid DNA IL-10F129S, 100 *μ*L at disease onset and 25 *μ*L 3 days later; ^8^1 mg/kg i.p. Rapamycin on D11, and every 2 days thereafter;^ 9^daily intrathecal 150 *μ*g ceftriaxone injection at symptom onset.

**Table 2:** Summary of pain symptoms observed in EAN models and potential therapeutic options for treating GBS pain.

EAN antigen	Animal model	Test site	Test	Symptoms during disease	Drug	Mode of action and effects of drug	References
200 *μ*g P2_(57–81)_	♂ Lewis rats	Forepaws & hindpaws	Hargreaves test	Thermal hyperalgesia (during disease course)			[115]
			Electronic von Frey	Mechanical allodynia (during disease course)			

80 *μ*g P2_(57–81)_	♂ Lewis rats	Hindpaw	Electronic von Frey	Mechanical allodynia (before clinical signs, during disease, and after recovery)			[116]

80 *μ*g P2_(53–78)_	♂ Lewis rats	Hindpaw	Electronic von Frey	Mechanical allodynia (during disease)	Minocycline (an antibiotic of tetracycline family)	Inhibition of expression of MMPs and infiltration of immune cells to PNS Significant decrease of disease severity Attenuate mechanical allodynia in EAN rats	[117]

100 *μ*g P2_(57–81)_	♂ Lewis rats	Hindpaw	Electronic von Frey	Mechanical allodynia (before clinical signs and during disease)	Compound A (a plant-derived glucocorticoid receptor ligand)	A selective glucocorticoid receptor ligand with anti-inflammatory action Attenuate neurological signs Reduce mechanical allodynia in rats	[118]

200 *μ*g P2_(57–81)_	♂ Lewis rats	Hindpaw	Dynamic plantar aesthesiometer	Mechanical allodynia (during disease and recovery)	CD28 superagonist (CD28SupA)	Expands regulatory T-cell population Ameliorate disease severity Reduction of mechanical pain hypersensitivity in rats	[119]

25 *μ*g bovine peripheral myelin	♀ Lewis rats	Hindpaw	Dynamic plantar aesthesiometer	Mechanical allodynia (before clinical signs)			[120]
